# Electrodeposition of Lithium-Based Upconversion Nanoparticle Thin Films for Efficient Perovskite Solar Cells

**DOI:** 10.3390/nano12122115

**Published:** 2022-06-20

**Authors:** Masfer Alkahtani, Hussam Qasem, Sultan M. Alenzi, Najla Alsofyani, Anfal Alfahd, Abdulaziz Aljuwayr, Philip R. Hemmer

**Affiliations:** 1 National Center for Renewable Energy, King Abdulaziz City for Science and Technology (KACST), Riyadh 11442, Saudi Arabia; hqasem@kacst.edu.sa (H.Q.); nalsofyani@kacst.edu.sa (N.A.); aalfahd@kacst.edu.sa (A.A.); jwr881@gmail.com (A.A.); 2 Institute for Quantum Science and Engineering, Texas A&M University, College Station, TX 77843, USA; prhemmer@tamu.edu; 3 National Center for Nanotechnology and Semiconductors, King Abdulaziz City for Science and Technology (KACST), Riyadh 11442, Saudi Arabia; sultan0064@gmail.com; 4 Department of Electrical and Computer Engineering, Texas A&M University, College Station, TX 77843, USA

**Keywords:** perovskite solar cell, upconversion nanoparticle thin films, lithium, efficiency

## Abstract

In this work, high-quality lithium-based, $LiYF_{4}=Yb^{3+},Er^{3+}$ upconversion (UC) thin film was electrodeposited on fluorene-doped tin oxide (FTO) glass for solar cell applications. A complete perovskite solar cell (PSC) was fabricated on top of the FTO glass coated with UC thin film and named (UC-PSC device). The fabricated UC-PSC device demonstrated a higher power conversion efficiency (PCE) of 19.1%, an additional photocurrent, and a better fill factor (FF) of 76% in comparison to the pristine PSC device (PCE = ~16.57%; FF = 71%). Furthermore, the photovoltaic performance of the UC-PSC device was then tested under concentrated sunlight with a power conversion efficiency (PCE) of 24% without cooling system complexity. The reported results open the door toward efficient PSCs for renewable and green energy applications.

## 1. Introduction

Lanthanide-doped upconversion nanoparticles (UCNPs) have recently attracted special interest in numerous interesting applications. Their ability to upconvert one or more low-energy NIR photons to one high-energy visible photon, as well as their photostability and photoluminescence tunability, opens the door for advanced and promising applications [1,2,3,4,5]. These include bio-applications, quantum sensing, optoelectronics, super-resolution microscopy, and renewable energy [6,7,8,9,10,11,12,13,14,15].

In solar cell applications, thin UC films are usually preferred when they are applied as spectral converters to capture sub-bandgap solar radiation. This technology has been implemented in silicon-based solar cells and showed a power conversion enhancement [16,17,18]. As the perovskite solar cells are considered to be the best alternative to the silicon-based solar cells, thin UC film technology applications in PSCs remains unexplored in detail. UCNP thin films are expected to enhance perovskite solar cells’ (PSCs) power conversion efficiency due to their exceptional near-infrared light (NIR) responsivity [10,11,12,13]. In addition, the UCNP thin films in PSCs will work as a spectral converter to harvest the near-infrared (NIR) solar photons from sunlight and convert them to absorbable visible light photons by the perovskite light-harvesting active layer [10,11].

In addition to upconversion, UNCPs can also down-convert UV light by a process known as quantum cutting giving more than one electron per photon and hence improved quantum efficiencies for UV illumination [19]. Also, UCNPs can act as a scattering layer, which can increase the light path [20].

To make this technology possible in PSCs, UCNP thin films were synthesized by several techniques such as sol-gel techniques [21], polymer matrix [22,23], or pulsed laser deposition (PLD) [24]. However, the thin films produced by these platforms suffer from low efficiency, instability, and expensive equipment, especially with the PLD technique. Recently, an efficient and inexpensive electrodeposition technique has been explored and developed to grow uniform $NaYF_{4}=Yb^{3+},Er^{3+}$, UC films with strong visible UC luminescence under a low NIR laser power excitation [25,26].

In this work, we electrodeposited lithium-based UC thin films, $LiYF_{4}=Yb^{3+},Er^{3+}$, on fluorene-doped tin oxide (FTO) glass for solar cell applications. The as-electrodeposited UC thin films were converted to pure hexagonal phase through a low-temperature annealing treatment, which leads to an intense visible (green and red) UC luminescence under a low-power NIR excitation. To probe the UC thin films’ application in the perovskite solar cells, a complete PSC device was fabricated and their performance was evaluated. The photovoltaic performance of the fabricated PSC on the UC thin films demonstrated an increase in PCE of 19.1% in comparison to pristine PSC devices. The reported results suggested an easy, efficient strategy to use efficient UC thin films to enhance the efficiency of PSCs for promising renewable energy applications.

## 2. Materials and Methods

### 2.1. Electrodeposition of $LiYF_{4}=Yb^{3+},Er^{3+}$ Thin Films

The electrodeposition of $LiYF_{4}=Yb^{3+},Er^{3+}$ films was conducted in an aqueous solution containing $Y^{3+}$ (78%), $Yb^{3+}$ (20%), $Er^{3+}$ (2%)-EDTA complexes, which were formed by stirring 0.011 M of EDTA and 0.01 M of $Ln{\left(NO_{3}\right)}_{3}$ solution for 30 min at pH 9 (pH was adjusted by adding few droplets of 1M of NaOH). After that, 0.05 M of $LiOH.H_{2}O$, 0.08 M of $Na_{4}F$ and 0.1 M sodium ascorbate were added subsequently, and the final pH of the solution was adjusted to 7 by concentrated $HNO_{3}$. Prior to deposition, the final solution was left under stirring for 5 min. For electrodeposition of UC thin films, FTO substrate was placed in the electrodeposition cell as a working electrode inside the electrodeposition solution prepared above. The UC thin films were electrodeposited at a temperature of 70 °C for 1 h under an applied anode potential of 0.8 V. The as-electrodeposited films were then annealed at 300 °C in air for 6 h in order to obtain a bright and pure hexagonal phase of $LiYF_{4}=Yb^{3+},Er^{3+}$ UC thin films.

### 2.2. Perovskite Solar Cell Devices Fabrication

Step 1. A thin compact TiO_2_ layer for all FTO substrates coated with UC thin films and without UC thin films (control PSC device) was prepared in a glove box filled with argon gas by spin-coating a solution containing 0.6 mL of titanium isoperopoxide, 0.4 mL of acetylacetonate, and 9.0 mL of ethanol onto the substrates at 2500 rpm for 30 s. The spin-coated layer was annealed at 450 °C for 30 min. After completing the annealing step, the mesoporous layer (150–200 nm) of TiO_2_ paste was then introduced on the top of the compact layer by spin-coating at 5000 rpm for 30 s and then was also annealed at 500 °C for 30 min.

Step 2. To prepare a perovskite precursor solution, in a glove box filled with argon gas, a solution of 18.84 mg of methylammonium bromide, 247.2 mg of formamidine bromide, 722.22 mg of lead(II) iodide, 62.04 mg of lead(II) bromide, 21.84 mg of cesium iodide, 960 µL of dimethylformamide (DMF), and 240 µL of dimethyl sulfoxide (DMSO) was mixed and then heated to 80 °C for 15 min to ensure homogeneity to obtain the triple-cation composition. Afterward, 50 µL of the precursor solution was spin-coated at two different rpm speeds at 1000 rpm for 10 s and then at 6000 rpm for 30 s, respectively. An amount of 200 μL of chlorobenzene was poured onto the substrates for 15 s and then the substrates were then annealed at 100 °C for 45 min on a hotplate to form crystalline triple-cation perovskite layers in order to remove residual DMSO and DMF in the precursor films.

Step 3. In a glove box filled with argon gas, a hole transfer layer (HT) was subsequently deposited on top of the triple cation perovskite layers by the spin-coating of a solution of N2,N2,N2′,N2′,N7,N7,N7′,N7′-octakis(4-methoxyphenyl)-9,9′-spirobi[9H-fluorene]-2,2′,7,7′-tetramine (spiro-MeOTAD) at 4000 rpm for 20 s.

Step 4. After that, an 80 nm-thick gold layer was thermally deposited on the top of the spiro-MeOTAD layers under a high vacuum using a special shadow mask. Finally, the fabricated PSC devices with an active area of 0.1 cm^2^ (0.25 × 0.4 cm^2^) were prepared for the photovoltaic performance measurements.

## 3. Results and Discussion

Experimentally, $LiYF_{4}=Yb^{3+},Er^{3+}$ UC thin films were electrodeposited following the modified procedure previously reported in [25]. Briefly, an aqueous solution contained 0.01 M of rare-earth ions $Ln{\left(NO_{3}\right)}_{3}$, where (Ln = $Y^{3+}$, $Yb^{3+}$, and $Er^{3+}$), and 0.011 M of EDTA were mixed and stirred for 30 min at pH = 9 to form a stable $RE^{3+}$-EDTA complex (see Material and Methods section for details). After that, 0.05 M of $LiOH.H_{2}O$, 0.08 M of $NH_{4}F$ and 0.1 M of sodium ascorbate ($C_{6}H_{7}O_{6}{}^{-}$) were added and the final pH of the solution was adjusted to a pH equal to 7 by adding a few drops of 0.01 M of $HNO_{3}$. Prior to deposition, the final solution was left under stirring for 5 min.

**Figure 1:**
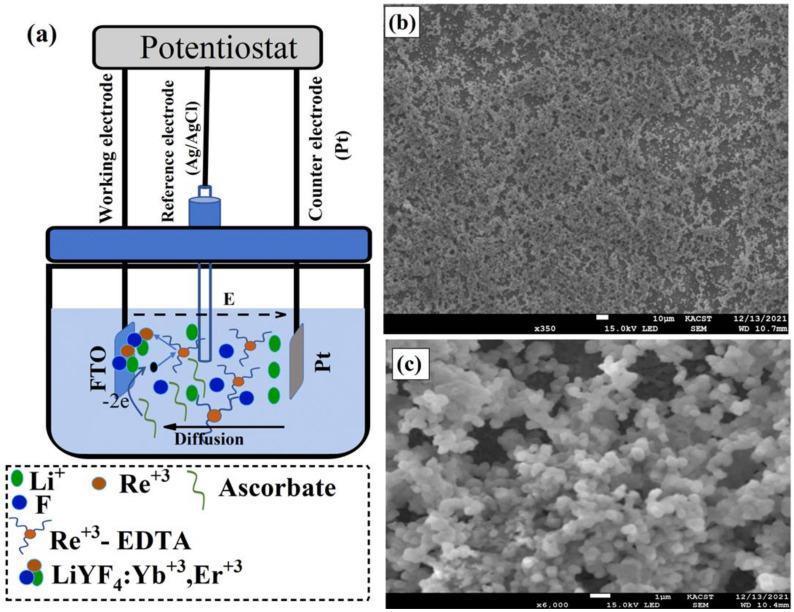
(**a**) Schematic illustration of lithium-based UC thin films, LiYF_4_: Yb, Er, electrodeposition process using Electrochemical Workstation. In the electrodeposition cells, a platinum plate (Pt) was used as a counter electrode, fluorine-doped tin oxide (FTO) glass as a working electrode, and an Ag/AgCl electrode as a reference electrode. Electrodeposition steps are also illustrated in Figure 1a where, $Li^{+}$, $Re^{3+}$, and $Re^{3+}$ -EDTA represents lithium ions, rare-earth elements, and rare-earth elements-EDTA complex, respectively. (**b**,**c**) Low- and high-magnification SEM images of LiYF_4_: Yb, Er films electrodeposited on FTO in aqueous solution at 70 °C and annealed at 300 °C for 6 h.

The UC thin films were electrodeposited using a conventional three-electrode cell connected to a computer-controlled electrochemical working station model CHI 601C Electrochemical Workstation (Shanghai, China), as illustrated in Figure 1a. In the electrodeposition cell, a platinum plate was used as a counter electrode, fluorine-doped tin oxide (FTO) glass with 20 Ω/${\mathrm{cm}}^{2}$ was used as a working electrode, and an Ag/AgCl electrode was used as a reference electrode. The FTO substrates that would later be used in the solar cell fabrication of the PSCs were cleaned in an acetone solution in an ultrasonic bath for 30 min and rinsed with distilled water (DI). After the cleaning procedure was complete, the FTO substrate was placed in the electrodeposition cell as a working electrode inside the electrodeposition solution prepared above. The UC thin films were electrodeposited at a temperature of 70 °C for 1 h under an applied anode potential of 0.8 V. The as-electrodeposited films were then annealed at 300 °C in air for 6 h in order to obtain the bright and pure hexagonal phase of $LiYF_{4}=Yb^{3+},Er^{3+}$ UC thin films.

The electrodeposition of $LiYF_{4}=Yb^{3+},Er^{3+}$ UC thin films involves different electrochemical reactions. EDTA acts as an electron-pair donor ligand for lanthanide ions to form a stable $RE^{3+}$-EDTA complex in an aqueous solution at a pH higher than 7. Under a positive applied potential, the released $H^{+}$ ions obtained through the oxidation of ascorbate and water will result in a lowering of the solution pH level and therefore cause a gradual dissociation of the $RE^{3+}$-EDTA complex into the free $RE^{3+}$ ions at the surface of the working electrode (FTO substrate). Finally, the dissociated $RE^{3+}$ ions around the FTO substrate will react and combine with $Li^{+}$ and $F^{-}$ to form $LiYF_{4}=Yb^{3+},Er^{3+}$ deposits on the working electrodes. For clarity, the formation of UC thin films can be explained by the following equations [25,26]:

(1) $$RE^{3+}+EDTA\;\to \;RE^{3+}-EDTA\;complex$$

(2) $$C_{6}H_{7}O_{6}{}^{-}\;\to \;C_{6}H_{7}O_{6}+H^{+}+2e\;$$

(3) $$H_{2}O\;\to \;\frac{1}{2}O_{2}+4H^{+}+2e$$

(4) $$RE^{3+}-EDTA+xH^{+}\;\to \;H_{x}EDTA_{x-4}+RE^{3+}$$

(5) $$RE^{3+}+4F^{-}+Li^{+}\;\to \;LiYF_{4}=Yb^{3+},Er^{3+}$$

**Figure 2:**
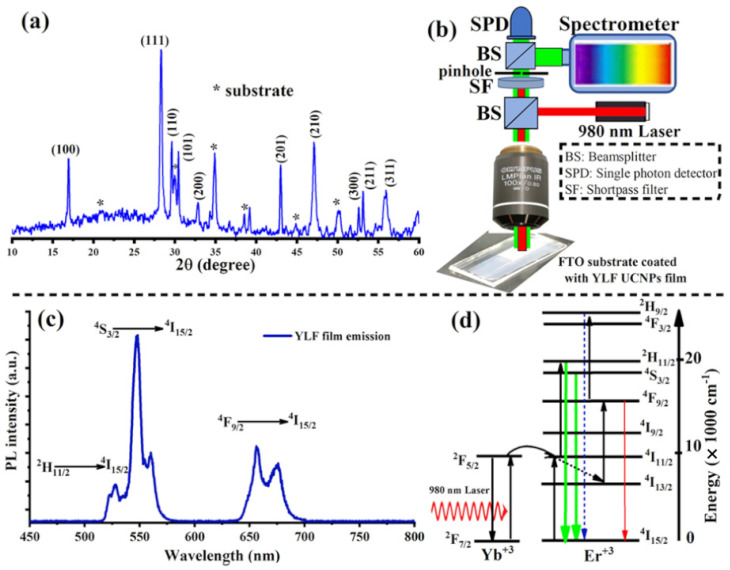
(**a**) XRD patterns of lithium-based UC thin films, LiYF4: Yb, Er, electrodeposited at 70 °C and annealed at 300 °C in air for 6 h. (**b**) An illustration of a home-made confocal microscope equipped with a 980 nm laser for photoluminescence (PL) measurement of electrodeposited UC thin films. (**c**) A strong UC emission spectrum measured directly from the electrodeposited UC thin films after air annealing. (**d**) The upconversion mechanisms of $Er^{3+}$ and $Yb^{3+}$ ions in the electrodeposited UC films under a 980 nm excitation.

To visualize the morphology of the electrodeposited UC thin films, the sample was placed in a scanning electron microscope (SEM). The UC films electrodeposited at 70 °C and annealed at 300 °C for 6 h in air show a uniform coating with aggregated spherical particles as shown in the low- and high-magnification The SEM images are illustrated in Figure 1c,d. The crystal quality of the deposited materials was then probed with an X-ray. Figure 2a shows the X-ray diffraction pattern (XRD) of the electrodeposited UC films, which revealed relatively sharp peaks, indicating crystalline, high-quality UC thin films.

The optical properties of the electrodeposited and annealed UC films were investigated. For this, we built a custom-made confocal laser-scanning microscope, equipped with continuous-wave (CW) 980 nm (NIR) lasers, an optical spectrometer, and a single-photon counter, as illustrated in Figure 2b. Under a relatively low intensity NIR laser excitation (20 W·cm^−2^), the UC films revealed a strong UC visible emission consisting of strong and sharp green peaks and relatively weak red peaks, as illustrated in Figure 2c. These emission peaks are the characteristic transitions of the $Er^{3+}$ ion according to the following optical transitions: H211/2→I415/2 (525 nm), S43/2→I415/2 (555 nm) and F49/2→I415/2 (654 nm), as illustrated in Figure 2d [5,10,14].

**Figure 3:**
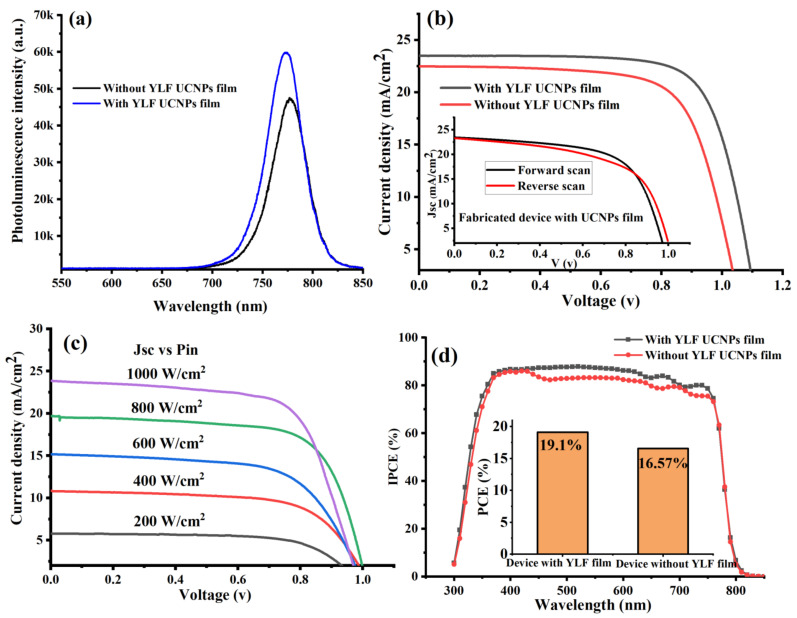
(**a**) Shows the photoluminescence spectra of perovskite films in PSCs with and without UC thin-film coating. (**b**) J-V characteristic curves measured under AM 1.5 G for fabricated PSCs with and without UC thin films. (**c**) A comparison chart of power conversion efficiency (PCE) of fabricated UC-PSC device and pristine device. (**d**) Quantum efficiency (IPCE) spectra of fabricated PSCs with and without UC thin films.

To explore the application of the electrodeposited UC thin films in perovskite solar cells, we fabricated a complete perovskite solar cell on top of the FTO glass coated with UC thin film named the UC-PSC device. For comparison, a control perovskite cell was then fabricated without UC thin film and named the pristine device. More details on how the PSCs’ layers were fabricated can be found in the Materials and Methods section. The optical emission spectra from the perovskite material with and without UC thin film were recorded and investigated under a green (532 nm) excitation using a confocal microscope. Under a green excitation at an intensity of 20 W·cm^−2^, the perovskite film in the UC-PSC device showed a strong emission peak at 780 nm, higher than that of the pristine film, as shown in Figure 3a. This observation could be attributed to the following explanations: first, that the grain boundaries were reduced by the electrodeposited UC thin films [27], second, that the non-radiative recombination was suppressed, and third, that the defect trap states were minimized [10,27].

Proceeding, we experimentally performed photocurrent density-voltage curves (J-V) under 1-sun illumination at AM 1.5 G to test the photovoltaic performance of the fabricated PSC devices. The data presented in Table 1 and Figure 3b, indicated that the electrodeposition of lithium-based UC thin films into the PSCs’ layers enhanced the photovoltaic performance. The UC-PSC device demonstrated a higher short-circuit current density (J_SC_) and power conversion efficiency (PCE), 4.25% and 14% enhancement, respectively, in comparison to the pristine device, as shown in Figure 3b,c. In addition, it was also observed that the open-circuit voltage (Voc) increased in the UC-PSC device in comparison to the pristine device. The enhancement in the photovoltaic performance of the UC-PSC device could be attributed to the additional photocurrent generated as a result of more incident NIR and UV photons being converted to visible photons by the UC films in the PSCs’ layers, and therefore, absorbed by the perovskite light-harvesting layer. Also, the high Li-ion content in the UC film is supposed to enable a faster electron transport within the mesoporous layer of the PSCs [27].

The anomalous hysteresis in the UC-PSC device illustrated in Figure 3b can be explained by the evoking of the charge migration or accumulation at the perovskite/ETL interface. The enhancement of the electrical properties and reduction in surface defects of ETL by a suitable UC thin-film coating can reduce the nonradiative recombination at the perovskite/ETL interface and thereby properly alleviate the anomalous hysteresis behavior of PSCs.

The light-intensity dependence of the J−V characteristics of the fabricated device (UC-PSC device) was measured under different incident light intensities ranging from 200 to 1000 W cm^−2^ (from 0.2 to 1 sun), as illustrated in Figure 3c. The J−V curves show almost a linear relation of the photocurrent on the light intensity, indicating no substantial space charge build-up in the integrated device.

The UC-PSC device showed a relatively higher incident photon-to-current conversion efficiency (IPCE) spectrum over the entire region of 300–800 nm in comparison to the pristine device as shown in Figure 3d. This broadband enhancement in the IPCE curve can be explained by the better charge carrier collection efficiency and lower charge recombination for the UC-PSC than those for the pristine device. In addition to enhancing electrical performance, YLiF_4_ UC thin films can improve long-term stability by surface passivation (YLF UC thin film/Perovskite interface) due to lattice matching, especially in the tetragonal phase.

The good photovoltaic performance of the UC-PSC device indicated the high quality of the electrodeposited UC films on the FTO substrate, which showed a mesoporous layer rather than a continuous layer in the SEM images reported in Figure 1. The UC film interlayer acts as a highly efficient interfacial passivation layer between the FTO/perovskite interface without influencing the surface topography and crystal quality of perovskite films as well as the light absorption ability of entire stack layers of the fabricated device.

**Figure 4:**
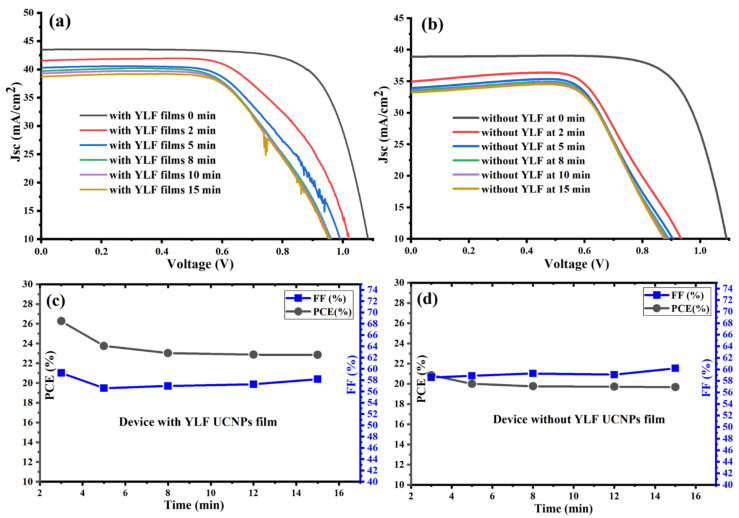
(**a**,**b**) J-V characteristic curves measured under concentrated AM 1.5 G for fabricated PSCs with and without UC thin films. (**c**,**d**) Power conversion efficiency (PCE) and fill factor (FF) of fabricated PSCs with and without UC thin films under a continuous concentrated light without cooling system complexity.

Next, we investigated the photovoltaic performance of the fabricated PSC devices vs. the time under concentrated illumination (plano-convex lens with a focal length equal to 150 mm). Figure 4a shows that the UC-PSC device gave a higher short-circuit current density (JSC) with an average value reaching (40 mA/cm^2^) under concentrated 1-sun irradiance and an average power conversion efficiency (PCE) reaching 24%, as shown in Figure 4b,c. A significant increase in Jsc and PCE values is expected as concentrated sunlight increases the generated current of a UC-PSC device as a function of the increased number of photons hitting the perovskite active layer [28].

Without an appropriate cooling system for the UC-PSC device, the dark current increases as a function of the operating temperature, resulting in reduced open-circuit voltage (Voc) for longer times, and therefore reducing the photovoltaic performance of the system [28,29], as illustrated in Figure 4a,c. In comparison to the UC-PSC device, the pristine device shows a relatively low photovoltaic performance, as illustrated in Figure 4b,d. The good photovoltaic performance of the UC-PSC device could be attributed to the better conversion efficiency of the more concentrated incident NIR photons to visible light photons. In addition, the Li-doping in the UC layer may enable a faster electron transport within the layers of the PSCs.

In summary, the UC-PSC device showed a good and stable photovoltaic performance under a continuous concentrated light without cooling system complexity, which opens the door for PSC integration into CPV systems for efficient solar cell applications.

## 4. Conclusions

We successfully electrodeposited lithium-based upconversion (UC) thin films, $LiYF_{4}=Yb^{3+},Er^{3+}$, on fluorene-doped tin oxide (FTO) glass in an aqueous solution at a low temperature. The UC optical properties of the as-electrodeposited thin films were activated under a low-temperature annealing treatment and showed an intense UC green luminescence under a NIR excitation. The use of these electrodeposited UC thin films was investigated in PSCs by fabricated PSCs directly on the UC thin film. These hybrid UC-PSC devices demonstrated an increase in PCE reaching 19.1% in comparison to pristine PSCs. The UC-PSC devices also demonstrated a higher and more stable photovoltaic performance under continuous illumination with concentrated light over a time period required to reach thermal equilibrium without a cooling system. These reported data show that UC thin films are a promising technology for producing more efficient, robust, and inexpensive PSCs in renewable energy applications.

## Figures and Tables

**Table 1:** Photovoltaic parameters of the fabricated devices.

Sample	Jsc (mA/cm^2^)	FF (%)	Voc (V)	PCE (%)
Pristine	21.49	71.3	1.084	16.57
UC-PSC device	23.01	76	1.118	19.1

## Data Availability

The data presented in this study are available on request from the corresponding author.
